# Whole-infrared-band camouflage with dual-band radiative heat dissipation

**DOI:** 10.1038/s41377-023-01287-z

**Published:** 2023-10-04

**Authors:** Bing Qin, Yining Zhu, Yiwei Zhou, Min Qiu, Qiang Li

**Affiliations:** 1 State Key Laboratory of Modern Optical Instrumentation, College of Optical Science and Engineering, Zhejiang University, Hangzhou, 310027 China; 2 Key Laboratory of 3D Micro/Nano Fabrication and Characterization of Zhejiang Province, School of Engineering, Westlake University, 18 Shilongshan Road, Hangzhou, 310024 Zhejiang China; 3 Institute of Advanced Technology, Westlake Institute for Advanced Study, 18 Shilongshan Road, Hangzhou, 310024 Zhejiang China

**Keywords:** Mid-infrared photonics, Metamaterials

## Abstract

Advanced multispectral detection technologies have emerged as a significant threat to objects, necessitating the use of multiband camouflage. However, achieving effective camouflage and thermal management across the entire infrared spectrum, especially the short-wave infrared (SWIR) band, remains challenging. This paper proposes a multilayer wavelength-selective emitter that achieves effective camouflage across the entire infrared spectrum, including the near-infrared (NIR), SWIR, mid-wave infrared (MWIR), and long-wave infrared (LWIR) bands, as well as the visible (VIS) band. Furthermore, the emitter enables radiative heat dissipation in two non-atmospheric windows (2.5–3 μm and 5–8 μm). The emitter’s properties are characterized by low emittance of 0.270/0.042/0.218 in the SWIR/MWIR/LWIR bands, and low reflectance of 0.129/0.281 in the VIS/NIR bands. Moreover, the high emittance of 0.742/0.473 in the two non-atmospheric windows ensures efficient radiative heat dissipation, which results in a temperature decrement of 14.4 °C compared to the Cr reference at 2000 W m^−2^ input power density. This work highlights the role of solar radiance in camouflage, and provides a comprehensive guideline for developing multiband camouflage compatible with radiative heat dissipation, from the visible to LWIR.

## Introduction

Camouflage refers to the ability to reduce the signal captured by detectors, thereby improving survival rates. However, the combination of detectors operating in multiple spectral bands poses a significant challenge^1,2^, necessitating the development of multiband camouflage technologies. On the one hand, objects in nature are illuminated by external light sources, the reflection of which can reveal their presence. Solar radiance is the primary natural light source during the daytime, which emits its energy mainly in the spectral range of 0.15–4 μm and plays a crucial role in the visible (VIS, 400–780 nm), near-infrared (NIR, 0.78–1.4 μm), and short-wave infrared (SWIR, 1.4–2.5 μm) detection bands. At night, artificial lighting sources such as searchlights and illuminating projectiles typically work in the visible and NIR bands to assist in object search. On the other hand, objects radiate energy through thermal emission^3,4^. The thermal emission in atmospheric transmission windows (typically mid-wave infrared (MWIR, 3–5 μm) and long-wave infrared (LWIR, 8–14 μm)) can be detected by infrared detectors. The intensity of a blackbody thermal emission is proportional to the fourth power of surface temperature, and the peak wavelength shifts to the short-wave direction as the temperature increases, making the radiative signal in the SWIR band non-negligible^5–7^. Additionally, reducing the surface temperature through radiative heat dissipation in non-atmospheric windows (undetected bands) can enhance the IR camouflage performance by mitigating heat load^8,9^.

Considerable efforts have been directed toward developing thermal camouflage using various techniques in the MWIR and LWIR bands, including metallic/dielectric structures^10–21^, electrochromic^22–29^ and thermochromic^30–33^ materials. Additionally, progress has been made in thermal camouflage through simultaneous radiative heat dissipation in the 5–8 μm non-atmospheric window using nano-structures (e.g., photonic crystals^34,35^, metal-insulator-metal metasurfaces^36–42^, Fabry-Perot cavities^43,44^, anti-reflection layers^43–45^, and porous nanostructures^46^). Besides, some studies have combined thermal camouflage with visible camouflage (e.g., cheating coloration^35,39,46^, transparency^37,41,44^, and low reflection^47^) or laser camouflage in the NIR band (by using metal-insulator-metal metasurfaces^38,39,42,48^ or photonic crystals^35^ to absorb or using coding metasurfaces^49^ to scatter the incident lasers) to address multiband detectors.

However, previous works have not adequately addressed several crucial aspects. Firstly, there has been a lack of attention paid to camouflage in the SWIR band, particularly in terms of countering thermal emission. With advancements in SWIR technologies, it has become increasingly important to develop SWIR camouflage after weighing external light and thermal emission in this band^50^. Secondly, the 2.5–3 μm non-atmospheric window is seldom used for radiative heat dissipation^44^, despite accounting for significant thermal energy emission as the object temperature increases. Thirdly, there has been insufficient focus on camouflage against broadband light sources in the VIS/NIR bands. These light sources are more general for objects and broadband camouflage could also complement laser camouflage in the VIS/NIR bands. Therefore, the manipulation of absorptance/emittance spectra covering the entire spectral range from VIS to LWIR, with efficient radiative heat dissipation, remains a significant challenge.

This work demonstrates the whole-infrared-band and visible-band camouflage with dual-band radiative heat dissipation using a lithography-free 7-layer structure. Firstly, low emissivity (0.270/0.042/0.218) in the SWIR/MWIR/LWIR bands is achieved, resulting in a substantial reduction of the signal in the SWIR band (39.3%, @ 200 °C) and significant radiative temperature decrements in the MWIR/LWIR bands (86.1/72.1 °C, @ 200 °C) compared to a blackbody reference. Additionally, outdoor experiments demonstrate the sample’s ability to achieve SWIR camouflage in sunshine. Secondly, low reflectivity (0.129/0.281) in the VIS/NIR bands is achieved to minimize the reflected signal. Thirdly, the high emissivity (0.742/0.473) achieved in two non-atmospheric windows (2.5–3 μm and 5–8 μm) results in a surface temperature decrease of 14.4 °C compared with conventional chromium film under an input power density of 2000 W m^−2^. This research offers opportunities for further development in spectrum manipulation for different bands and scenarios against complicated signal sources and multispectral detective technologies. A detailed comparison of infrared and visible camouflage with existing camouflage materials and structures can be found in Table S1, Supplementary Information.

## Results

### Principle for whole-infrared-band and visible-band camouflage

**Fig. 1:**
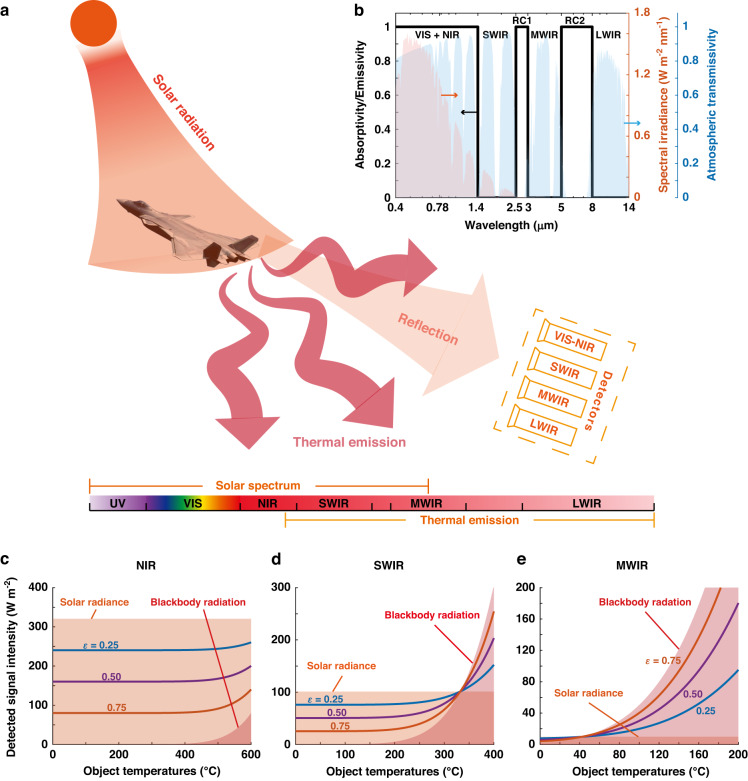
Principle for whole-infrared-band and visible-band camouflage compatible with radiative heat dissipation. **a** Typical detection bands ranging from visible to long-wave infrared and two primary signal sources: the reflection of solar radiation and thermal emission of the object. **b** The absorptivity/emissivity spectrum (black line) of an ideal wavelength-selective emitter designed to counter multiband detectors. The red and blue areas represent the solar irradiance spectrum and atmosphere transmittance spectrum, respectively. **c**–**e** The band-integrated irradiance of solar radiance and blackbody radiation at various object temperatures in the NIR, SWIR, and MWIR bands. The total detected signal intensity of objects with different average emissivity (*ε* = 0.25, 0.5, 0.75) are plotted in solid lines

Objects typically betray their presence through two types of signals: reflected signals from external light sources and thermal emission signals from the objects themselves (Fig. 1a). The sum of these signals, as expressed in Eq. (1), represents the total detected signal intensity. Solar radiance is the most significant natural external light source, and its impact on camouflage is particularly pronounced in the VIS, NIR, and SWIR bands. Earth radiation is another significant natural external light source, which has impacts mainly in the LWIR band. The first term on the right-hand side of Eq. (1) represents the intensity of the reflected signal, which is obtained by integrating the spectral irradiance of solar radiance, *I*_solar_, and earth radiation, *I*_earth_, over the detection band (*λ*1 − *λ*2) and multiplying by the object’s reflectivity, *r*. The second term represents the intensity of the emission signal, which is obtained by integrating the spectral irradiance of a blackbody, *I*_BB_, over the detection band and multiplying by the object’s emissivity, *ε*. Kirchhoff’s law and the energy conservation law dictate that *r* is equal to 1 − *ε*, since the transmissivity is zero.

1 $$\begin{array}{l}{\rm{Signal}}\,{\rm{Intensity}}=\mathop{\int }\nolimits_{\lambda 1}^{\lambda 2}r({I}_{{\rm{solar}}}(\lambda )+{I}_{{\rm{earth}}}(\lambda ))d\lambda\\\qquad\qquad\qquad\quad\;\;\; +\mathop{\int }\nolimits_{\lambda 1}^{\lambda 2}\varepsilon {I}_{{\rm{BB}}}(\lambda ,{T}_{{\rm{object}}})d\lambda\end{array}$$

In the SWIR band, both external light (i.e., sunshine) reflection and thermal emission can be dominant (Fig. 1d), making SWIR camouflage challenging. Under clear weather conditions, the highest irradiance of sunshine can reach ~100 W m^−2^, which is similar to that of a 330 °C blackbody. This temperature (330 °C) can be considered a critical temperature, and inhibiting thermal emission becomes more significant if the object temperatures are above it (Fig. 1d). In practical scenarios, where solar irradiance is generally lower than ideal conditions, this critical temperature will decrease considerably, making low emissivity more suitable for SWIR camouflage across a broader temperature range (as demonstrated in Supplementary 1).

In the MWIR and LWIR ranges, thermal emission usually dominates the detected signal, as the intensity of sunshine is weak enough to be negligible (Fig. 1e). For example, in the MWIR band, solar irradiance is only about 10 W m^−2^, and objects with lower emissivity (i.e., *ε* = 0.25) exhibit smaller total signal intensity, as thermal emission is significantly suppressed (Fig. 1e). In the LWIR band, the earth radiation will have impacts on the camouflage of the air targets. But in general cases, the temperatures of the air targets are higher than that of the earth, and suppressing thermal emission is an effective way to reduce the total signal intensity (see Supplementary 2). Therefore, low emissivity is more suitable for camouflage in the MWIR and LWIR bands.

In the VIS and NIR ranges, the predominant source of detected signal is reflected solar radiance or other natural/artificial light, as the thermal emission from objects is generally insignificant (Fig. 1c). The integrated solar irradiance in the NIR band is ~320 W m^−2^, whereas the integrated blackbody irradiance is less than 20 W m^−2^ up to a temperature of 500 °C. Objects with lower reflectivity (i.e., higher emissivity, *ε* = 0.75), exhibit a smaller total signal intensity within the temperature range of 0–600 °C (Fig. 1c). Therefore, low reflectivity is preferable for effective camouflage in the VIS and NIR bands.

In summary, the spectral features of objects need to meet several criteria (represented by the black line in Fig. 1b): (1) low emissivity in the SWIR, MWIR, and LWIR bands to suppress thermal emission; (2) low reflectivity in the VIS and NIR bands to reduce the reflected signals; (3) high emissivity in non-atmospheric windows (2.5–3 μm and 5–8 μm ranges) to enable efficient radiative heat dissipation. These criteria are essential for achieving whole-infrared-band camouflage with dual-band radiative heat dissipation and can guide the design of camouflage materials and structures.

### Structure design and measurements

**Fig. 2:**
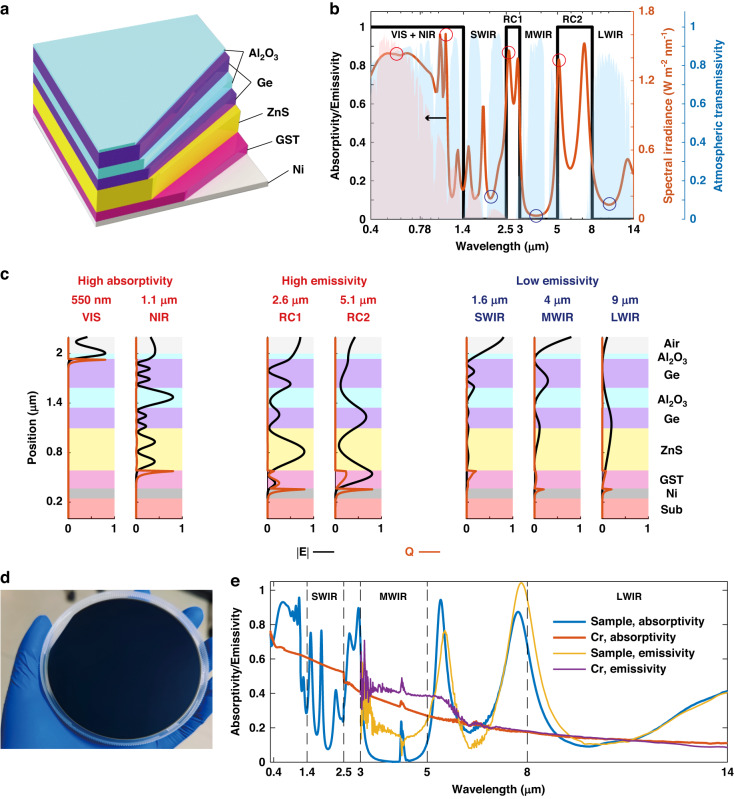
Simulations and characterizations of the camouflage structure that enables compatibility with whole infrared and visible bands. **a** The multilayer structure utilized for camouflage. **b** The simulated absorptivity spectrum of the structure. **c** The electric field intensity |E| (black lines) and resistive loss Q (orange lines) distribution at various typical wavelengths in high absorptivity/emissivity bands (red circles in the spectrum) and low emissivity bands (blue circles in the spectrum). **d** The designed multilayer structure is deposited on a 4-inch silicon substrate. **e** The measured absorptivity spectrum (300 nm–14 μm) and emissivity spectrum (3–14 μm, @ 150 °C) of the sample and the Cr reference

The Al_2_O_3_(65 nm)/Ge(350 nm)/Al_2_O_3_(240 nm)/Ge(250 nm)/ZnS(510 nm)/GST(220 nm)/Ni(120 nm) multilayer structure is employed to modulate the ultra-broad spectrum from the visible to the LWIR range (Fig. 2a). The unique architecture of this structure allows it to cater to the varying demands of the entire infrared range and the visible range, while concurrently achieving efficient radiative heat dissipation within two non-atmospheric windows. The bottom GST/Ni layers function as an anti-reflection structure in non-atmospheric windows (Fig. S4a). The crystalline GST, with a high refractive index and a high extinction coefficient, facilitates the reduction of the overall thickness of the structure and contributes to the high emissivity in two radiative heat dissipation bands. The center Ge/Al_2_O_3_/Ge/ZnS layers help to optimize the spectrum in the SWIR and 2.5–3 μm band by compensating for the refractive index change of GST in these bands (Fig. S4b). The top Al_2_O_3_ layer mitigates the reflection (i.e., enhances the absorption) in the VIS and NIR bands (Fig. S4c).

The multilayer structure’s absorptivity/emissivity spectrum is simulated (Fig. 2b), and the electric field intensity |E| and resistive loss Q distribution are presented (Fig. 2c). In the VIS band (*λ* = 550 nm in Fig. 2c), the Ge layer located beneath the Al_2_O_3_ anti-reflection layer is opaque, leading to rapid electric field attenuation. In the NIR range (*λ* = 1.1 μm), the GST layer becomes opaque, where resistive loss mainly occurs. In the SWIR, MWIR, and LWIR bands (*λ* = 1.6, 4, 9 μm, respectively), both the electric field intensity and the resistive loss are inhibited, resulting in low emissivity in these bands. In the two radiative heat dissipation bands (*λ* = 2.6 μm and *λ* = 5.1 μm), the light can propagate through the GST layer and finally attenuate in the Ni layer, with resistive loss occurring in both the GST and Ni layers.

The multilayer structure is experimentally deposited onto a 4-inch silicon substrate (Fig. 2d), and a 240-nm-thick Cr film is employed as a reference on a similar substrate for its extensive use in the coating of objects^51,52^. The measured absorptivity spectrum (300 nm–14 μm) and the emissivity spectrum (3–14 μm, @ 150 °C) are shown in Fig. 2e. The measured absorptivity spectrum, which is equivalent to the emissivity spectrum according to Kirchhoff’s law, is found to be in good agreement with the measured emissivity spectrum. The deviation in the 3–6 μm range is attributed to the noise, as the temperature is only 150 °C.

In the SWIR/MWIR bands, the average emissivity of the sample is measured to be 0.270/0.042, respectively, which is lower than that of the Cr reference (0.530/0.306). In the LWIR band, the average emissivity of the sample (0.218) is slightly higher than the Cr reference (0.133). Moreover, in the two radiative heat dissipation bands (2.5–3 μm and 5–8 μm), the sample exhibits a high average emissivity of 0.742 and 0.473, respectively, highlighting its exceptional radiative heat dissipation performance.

In the VIS/NIR bands, the sample demonstrates an average reflectivity of 0.129/0.281, resulting in minimal reflections when illuminated. In the conventional silicon detector response wave band (0.3–1 μm), the sample exhibits a reflectivity of 0.151, which is lower than that of the Cr reference (0.333). Notably, the sample reflects more short-wave light (blue and UV light), resulting in a dark blue appearance to the naked eye (Fig. 2d). In addition, the multilayer structure can change its color to blend in with various environments by varying the thickness of the top Al_2_O_3_ layer (see Supplementary 5). Thus, the spectral characteristics of the sample are efficient for VIS/NIR camouflage, particularly in a dim environment.

### MWIR/LWIR camouflage and radiative heat dissipation

**Fig. 3:**
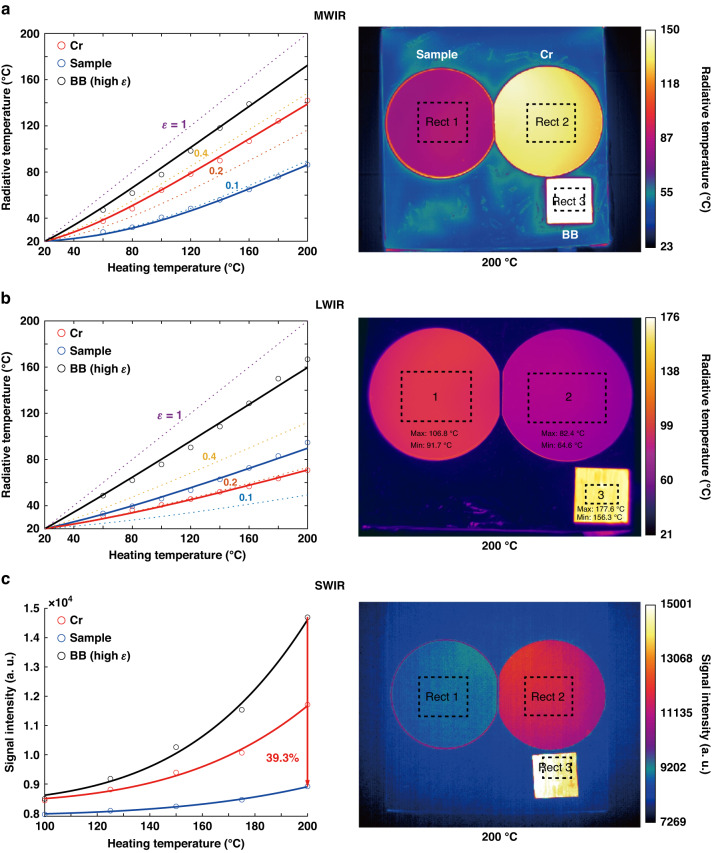
Demonstration of the infrared camouflage (SWIR, MWIR, and LWIR). The sample is placed on the heat stage, along with the Cr reference and blackbody reference (BB), and was heated up to 200 °C. **a**, **b** The radiative temperature variation of the sample/Cr/BB and the thermal images within the MWIR and LWIR bands. **c** The radiative signal intensity variation of the sample/Cr/BB under different heating temperatures within the SWIR band, and the thermal image at 200 °C. The experimental radiative temperatures (represented by circles in the left figures) were calculated by averaging the values in the rectangle areas in the thermal images obtained at various heating temperatures. The dashed lines represent the numerical calculations of radiative temperatures for various band average emissivity (0.1, 0.2, 0.4, and 1)

In order to evaluate the camouflage effect of the sample in the MWIR/LWIR bands, thermal imagers are utilized to capture the IR signals emitted by the sample, along with the Cr reference and a blackbody reference. The captured signal includes the thermal emissions of the objects as well as the reflected thermal emissions of the environment (with a temperature of 20 °C), including the walls and roof of the room. The results obtained in the MWIR band (Fig. 3a) indicate that the sample demonstrates a remarkable decrease in signal intensity in comparison to the Cr reference and the blackbody reference. Specifically, at 200 °C, the sample’s radiative temperature in the MWIR band is 86.3 °C, which is 55.7 °C lower than that of the Cr reference and 86.1 °C lower than that of the blackbody reference. In the LWIR band (Fig. 3b), the sample’s radiative temperature is 94.7 °C at 200 °C, which is 24 °C higher than that of the Cr reference but substantially lower than that of the blackbody reference (by ~72.1 °C). Therefore, the sample demonstrates camouflage performance that is comparable to or better than that of the Cr reference in the MWIR/LWIR bands.

Radiative temperatures in the MWIR and LWIR bands are calculated numerically using Eq. (2), which incorporates parameters such as *I*_BB_ (spectral irradiance of a black body), *λ*1/*λ*2 (minimum/maximum wavelength of the measuring band), *T*_radiative_ (object’s radiative temperature), *T*_actual_ (object’s actual temperature), *T*_environment_ (environment temperature), *ε* (emissivity of the object), and *r* (reflectivity of the object). The dashed lines presented in the left part of Fig. 3a, b (curves of *ε* = 0.1, 0.2, 0.4, and 1) are used to compare the calculated results with the experimental data, and to assist in estimating the emissivity of the sample and Cr reference:

2 $$\begin{array}{l}\mathop{\int }\nolimits_{\lambda 1}^{\lambda 2}{I}_{{\rm{BB}}}(\lambda ,{T}_{{\rm{radiative}}})d\lambda =\mathop{\int }\nolimits_{\lambda 1}^{\lambda 2}\varepsilon {I}_{{\rm{BB}}}(\lambda ,{T}_{{\rm{actual}}})d\lambda\\\qquad\qquad\qquad\qquad\qquad\;\; +\mathop{\int }\nolimits_{\lambda 1}^{\lambda 2}r{I}_{{\rm{BB}}}(\lambda ,{T}_{{\rm{environment}}})d\lambda\end{array}$$

**Fig. 4:**
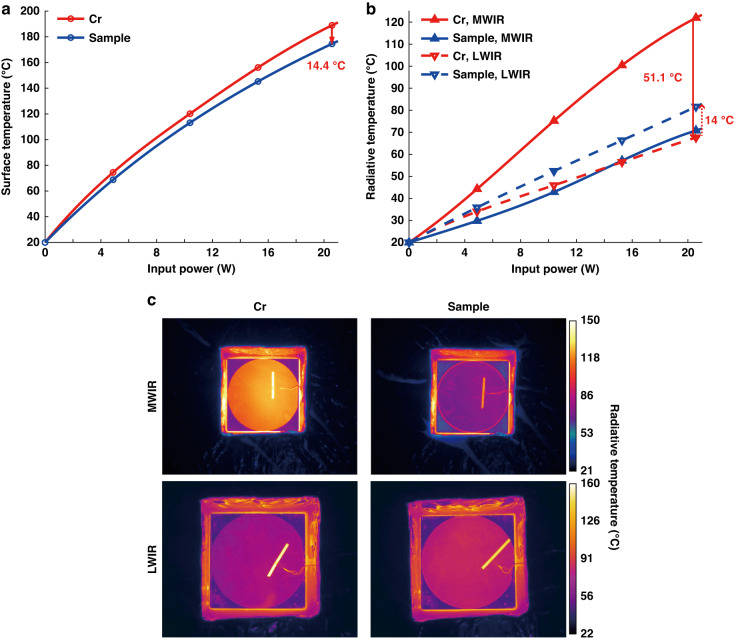
The improved MWIR/LWIR camouflage achieved through radiative heat dissipation. The 4-inch-size sample and Cr reference are placed in a polystyrene box to inhibit thermal conduction and convection and heated under the constant input powers. **a** The measured surface temperatures of the sample/Cr reference as the input power increases from 0 W to 20 W. **b** The radiative temperatures variation of the sample/Cr reference within the MWIR and LWIR bands. **c** The MWIR and LWIR band thermal images of the sample and Cr reference when the input power is 20 W

In order to demonstrate the efficacy of radiative heat dissipation, surface temperatures of the sample and the Cr reference are measured under constant input powers (Fig. 4a). The sample with a heat plate is placed inside a polystyrene box, which effectively suppresses thermal conduction and convection while allowing thermal radiation through a cubic window (see Supplementary 6). The high emissivity of the sample in two radiative heat dissipation bands allows for lower surface temperatures compared to the Cr reference when subjected to the same input electrical heating powers (Fig. 4a). At 20 W input power (equivalent to a power density of 2000 W m^−2^), the surface temperature of the sample is 174.5 °C, which is 14.4 °C lower than that of the Cr reference. These lower surface temperatures help to reduce thermal load and improve the performance of MWIR and LWIR camouflage.

The improved camouflage performance of the sample in the MWIR/LWIR bands is demonstrated using radiative temperatures measured by thermal imagers (Fig. 4b, c). In the MWIR band, the radiative temperature of the sample is 51.1 °C lower than that of the Cr reference with 20 W power input (Fig. 4b). This difference is more significant than that observed under the same surface temperature conditions (which is 47 °C, Fig. 3a). In the LWIR band, the sample’s radiative temperature is 14 °C higher than the Cr reference with 20 W power input (Fig. 4b), while the difference increases to 16.8 °C under the same surface temperature condition (Fig. 3b). Thus, radiative heat dissipation benefits the MWIR/LWIR camouflage performance of the sample.

### SWIR camouflage without/with sunshine

To examine the sample’s SWIR camouflage capability against thermal emission, experiments are first conducted indoors to emulate a scenario with no external light sources. The objects (i.e., the sample, the Cr reference, and the blackbody reference) are heated up and their signal intensities are measured using a thermal imager operating in the spectral range of 1.5–2.5 μm (Fig. 3c). As the objects’ temperatures rise from 100 to 200 °C, the blackbody reference exhibits a 73% increase in signal intensity while the sample’s signal intensity only increases by 12% due to its low emissivity in the SWIR band. At 200 °C, the sample demonstrates superior SWIR camouflage performance against thermal emission by exhibiting a 23.9% and 39.3% reduction in signal intensity compared to the Cr reference and the blackbody reference, respectively.

**Fig. 5:**
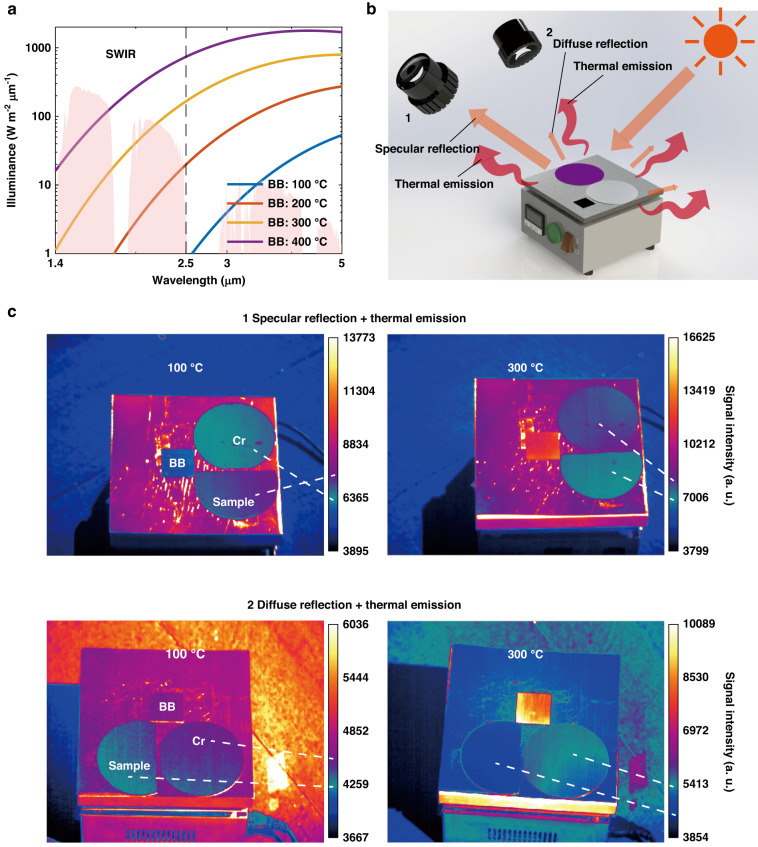
Camouflage in SWIR band with sunshine. **a** The spectral illuminance of solar radiation and black bodies at various temperatures in the IR bands. **b** A typical scenario for IR camouflage with sunshine. In the specular reflection direction of sunlight (1), strongly reflected light and thermal emission can be captured by thermal imagers. However, in other directions (2) only weak diffused light and thermal emission can be captured. **c** Thermal images captured in the SWIR band in directions 1 and 2 at 100 °C and 300 °C, respectively

In practical outdoor scenarios, sunlight reflection must be taken into account when designing SWIR camouflage, as the highest irradiance of sunlight in the SWIR band can rival that of a 300 °C blackbody (Fig. 5a). Two directions, namely, direction 1 with strong specular reflection of sunlight and omnidirectional thermal emission, and direction 2 with weak diffused reflection and thermal emission, are then evaluated for SWIR camouflage under sunlight (Fig. 5b). The SWIR camouflage at different object temperatures and observing directions are demonstrated with thermal imagers (Fig. 5c). At lower temperatures (e.g., 100 °C), the specular reflection of sunlight is stronger than the thermal emission. Consequently, in direction 1, the sample with higher reflectivity exhibits a stronger signal intensity than the Cr reference and blackbody reference. However, in direction 2, where the diffused reflection is weak, the sample with lower emissivity has a weaker signal intensity in thermal images. At higher temperatures (e.g., 300 °C), thermal emission is stronger than sunlight reflection (for both specular and diffused reflection cases). As a result, in both directions 1 and 2, the signal intensity of the sample remains weaker than the Cr reference and blackbody reference. Therefore, the sample demonstrates better SWIR camouflage performance, except in the specular direction at lower temperatures.

## Discussion

A multilayer wavelength-selective emitter is demonstrated to enable whole-infrared-band and visible-band camouflage compatible with dual-band radiative heat dissipation. Compared with the previous study on infrared and visible camouflage, the design is much simpler and possesses more challengingly compatible features (Table S1). Firstly, signal reduction in the SWIR, MWIR, and LWIR bands is achieved through the use of low emissive surfaces, while high absorption in the VIS and NIR bands is employed to minimize reflective signal. Secondly, efficient radiative heat dissipation is achieved in two non-atmospheric windows (2.5–3 μm and 5–8 μm), which satisfies thermal management requirements and improves MWIR/LWIR camouflages. Thirdly, a lithography-free structure with only seven layers is used to achieve ultra-broad band spectrum manipulation, providing potential for large-area applications. Finally, this device for whole-infrared-band camouflage can facilitate applications that require sophisticated spectrum manipulation, while balancing complex and contradictory requirements. Ultimately, this work is anticipated to stimulate innovative avenues for modern thermal management technologies and contribute to an energy-efficient future^3,8,9,17,53–55^.

## Materials and methods

### Simulation

The simulation of the absorptivity/emissivity spectrum was accomplished by leveraging the transfer matrix method and FDTD Solutions. Additionally, FDTD Solutions was employed to simulate the distribution of the electric field intensity. The refractive index and extinction coefficient of the materials utilized in the simulations are provided in Supplementary 3.

### Fabrication

The deposition of Ni and GST layers was carried out on a 4-inch silicon substrate using the magnetron sputtering technique. The deposition process was followed by the deposition of an Al_2_O_3_/Ge/Al_2_O_3_/Ge/ZnS multilayer film using the E-beam evaporation method. Additionally, the reference Cr film was also deposited using the magnetron sputtering method.

### Optical characterization

The spectral range spanning from 0.3 to 1.1 μm was probed for reflectivity using a spectrophotometer (Agilent Cary7000). The reflectivity spectrum in the spectral range of 1.1 to 14 μm was acquired by exploiting an FTIR microscope (Hyperion 1000, Brucker) and an FTIR spectrometer (Vertex 70, Brucker), while utilizing an MCT detector. For emissivity measurements, the FTIR spectrometer was employed, in conjunction with sample heating to 150 °C, to probe the spectral range of 3 to 14 μm.

### Radiative intensity/temperature and absolute temperature measurements

The radiative temperature was measured using specific equipment in different spectral bands. A Jenoptik camera was employed to measure the radiative temperature in the LWIR band. A Telops camera was used to measure the radiative temperature in the MWIR band and the signal intensity in the SWIR band (with a 1.5–2.5 μm band-pass filter). The absolute surface temperature was measured using thermocouples (5TC-TT-K-30-36, Omega) under normal pressure conditions.

### Supplementary information

Supplementary
