# Supplementary material for: Whole-infrared-band camouflage with dual-band radiative heat dissipation
Source: Light Sci Appl. 2023 Oct 4;12:246. doi: 10.1038/s41377-023-01287-z (PMC10550919; doi:10.1038/s41377-023-01287-z)
Supplement: Supplementary file 1 — Supplementary [file 41377_2023_1287_MOESM1_ESM.docx]

Supplementary Information for

**Whole-infrared-band camouflage with dual-band radiative heat dissipation**

*Bing Qin^1^, Yining Zhu^1^, Yiwei Zhou^1^, Min Qiu^2,3^, and Qiang Li^1,*^*

^1^State Key Laboratory of Modern Optical Instrumentation, College of Optical Science and Engineering, Zhejiang University, Hangzhou 310027, China

^2^Key Laboratory of 3D Micro/Nano Fabrication and Characterization of Zhejiang Province, School of Engineering, Westlake University, 18 Shilongshan Road, Hangzhou 310024, Zhejiang Province, China

^3^Institute of Advanced Technology, Westlake Institute for Advanced Study, 18 Shilongshan Road, Hangzhou 310024, Zhejiang Province, China

^*^E-mail: qiangli@zju.edu.cn

**Supplement 1. The detected signal intensity with various solar irradiance**

Solar radiance is a crucial natural light source outdoors, with its spectral coverage extending from ultraviolet to infrared. The visible (VIS) and near-infrared (NIR) bands constitute 52% and 32% of the solar radiance energy, respectively, making them significant signal sources that overwhelm the thermal emission signal of objects. Although occupying only 10% of the solar radiance energy, the short-wave infrared (SWIR) band's sunshine irradiance in satisfactory weather conditions is comparable to that of 330 ℃ blackbody radiance. However, solar radiance intensity strongly correlates with solar altitude and weather conditions. Moreover, the reflective conditions of the object, such as specular or diffused reflection, affect the final reflected signal that enters the detectors. The detected signal intensity of objects with different emissivities (*ε* = 0.25, 0.5, and 0.75) was computed using equation (1) when solar irradiance reduced to 60%, 30%, and 0% of the highest. In the NIR band, solar radiance remains dominant, albeit with a 30% reduction in intensity. In the absence of sunshine, thermal emission is negligible under 400 ℃ since it is relatively weak. However, in the SWIR band, the critical temperature decreases from 330 ℃ to 270 ℃ as the solar irradiance reduces to 30%, making thermal emission more dominant in the detected signal over a broader temperature range. When the solar irradiance varies from 100% to 5% of the highest level, the critical temperature in the NIR band decreases from 704 ℃ to 504 ℃ (Fig. S1c), and the critical temperature in the SWIR band decreases from 332 ℃ to 199 ℃ (Fig. S1d).

Fig. S1 | Signal intensity in the NIR/SWIR bands under various solar irradiance conditions. In a, b, the detected signal intensity in the NIR/SWIR bands is shown when solar irradiance reduces to 60%, 30%, and 0% of the highest level. The decrease in solar irradiance can be attributed to diverse factors, such as the solar, weather, and reflection conditions, leading to suboptimal conditions for signal detection. The detected signal is composed of both the reflection of solar radiance and thermal emission of the object. To investigate the effects of the emissivity (*ε* = 0.25, 0.5, and 0.75) on the signal intensity of different objects, their respective signal intensities were plotted. In c, d, the critical temperature variation in the NIR/SWIR band is shown when solar irradiance varies from 100% to 5% of the highest level.

**Supplement 2. The detected signal intensity with various earth irradiance**

In the LWIR band, the earth radiation will have impacts on the camouflage of the air targets. The intensity of the earth radiation is related to the temperature of the earth. As the temperature of the earth increases from 0 ℃ to 40 ℃, the earth irradiance will rise from 110 W m^-2^ to 208 W m^-2^ (Fig. S2). However, in general cases, the temperatures of the air targets are higher than that of the earth, and thus objects with a lower emissivity (e. g. *ε* = 0.25) will exhibit smaller total signal intensity. Therefore, suppressing thermal emission is an effective way to reduce the total signal intensity in the LWIR band.

Fig. S2 | Signal intensity in the LWIR band with various earth irradiance. The earth irradiance rises from 110 W m^-2^ to 208 W m^-2^ as the temperature of the earth increases from 0 ℃ to 40 ℃.

**Supplement 3.** **Refractive index and extinction coefficient of the materials**

**Fig. S3 | Refractive index (n) and extinction coefficient (k) of the materials.**

**Supplement 4.** **The working mechanism behind the structure design**

**Fig. S4 | Simulated spectra of various layer combinations. a** The absorptivity, reflectivity, and transmissivity spectrum of GST/Ni layer combination. **b** The absorptivity, reflectivity, and transmissivity spectrum of Ge/Al_2_O_3_/Ge/ZnS layer combination. **c** The absorptivity spectrum of the multilayer structure with/without the top Al_2_O_3_ layer.

**Supplement 5.** **Reflectivity spectrum variation with different thicknesses of the top Al_2_O_3_ layer**

**Fig. S5 | Reflectivity spectrum variation with different thicknesses of the top Al_2_O_3_ layer. a** Reflectivity spectra for different thicknesses of the top Al_2_O_3_ layer varying from 20 nm to 400 nm. **b** The corresponding visible colors indicated in the CIE 1931 chromaticity diagram.

**Supplement 6. Setup for radiative heat dissipation demonstration experiments.**

The experimental setup consisted of a main body apparatus made of expanded polystyrene (EPS) measuring 30×30×12 cm^3^. A cuboid shape measuring 12×12×6 cm^3^ was removed from the middle of the upper portion of the box to create a window. Both the inner and outer surfaces of the box were covered with aluminum foil. The heat plate was then placed inside the box and a piece of aerogel was positioned beneath it. The sample/Cr reference was carefully placed on the heat plate, and to suppress air convection, a polyethylene (PE) film was used to cover the upper surface of the box. A constant power source was used to supply the heat plate while a thermocouple was attached to the surface of the sample/Cr reference to measure temperature. The input power is calculated by Joule’s law: *P = UI*. The size of the heat plate is 10×10 cm^2^, and the average power density is calculated by *P*_density_ *= P / A*.

**Fig. S6 |** **Setup for radiative heat dissipation demonstration experiments.**

**Supplement 7. The radiative heat dissipation contributions of the two non-atmospheric windows**

Two non-atmospheric windows are utilized for radiative heat dissipation, which helps mitigate the thermal load of the objects and improve infrared camouflage performance, particularly for high-temperature objects. At 300 ℃, 93 W m^-2^ thermal energy is radiated through the 2.5 - 3 μm band and 891 W m^-2^ energy is dissipated through the 5 - 8 μm band (Fig. S7a). As the object temperature rises, radiative heat dissipation becomes increasingly significant, particularly through the 2.5 - 3 μm band (Fig. S7b). At 500 ℃, the power dissipated through radiative channel reaches 979 W m^-2^ / 2628 W m^-2^ in the 2.5 - 3 μm / 5 - 8 μm band. Furthermore, the proportion that the 2.5 - 3 μm band occupies in the total energy dissipated increases from 9.45% to 27.14% as the temperature rises from 300 ℃ to 500 ℃.

**Fig. S7 | The radiative heat dissipation contributions of the two non-atmospheric windows.** **a** The spectral illuminance of the blackbody (black line) and the wavelength-selective emitter (orange line) at 300 ℃. **b** The power density variation of radiative heat dissipation in the two non-atmospheric windows when the object temperature increases.

**Supplement 8. Comparison of infrared and visible camouflage**

**Table S1. | Comparison of infrared and visible camouflage.**

| Structure | | Multiband camouflage | | | | | | | | | | Radiative heat dissipation | |
| --- | --- | --- | --- | --- | --- | --- | --- | --- | --- | --- | --- | --- | --- |
|  |  | VIS | | | | NIR | SWIR | MWIR | LWIR | | | 2.5-3 μm | 5-8 μm |
| 2-D gratings^1^ | | Colorful | | | / | | / | Low ε (0.25) | Low ε (0.33) | | | / | High ε (0.77) |
| 1-D photonic crystals^2^ | | Colorful | | | Δ | | / | Low ε (0.11) | Low ε (0.12) | | | / | High ε (0.61) |
| Metal-insulator-metal metasurface^3^ | | Trans. | | | / | | / | Low ε | Low ε | | | / | High ε |
| Ring & disk metasurface^4^ | | / | | | Δ | | High abs.* | Low ε | Low ε | | | / | High ε |
| Porous nanostructured composite film^5^ | | Colorful | | | / | | / | Low ε (0.10) | Low ε (0.21) | | | / | High ε |
| Coding metasurface^6^ | | / | | | Δ | | / | Low ε (0.19) | Low ε (0.11) | | | / | / |
| Metal-semiconductor-metal metasurface^7^ | | Colorful | | | Δ | | High abs.* | Low ε | Low ε | | | / | / |
| Metal-insulator-metal metasurface^8^ | | Trans. | | | / | | / | Low ε (0.24) | Low ε (0.09) | | | / | High ε (0.57) |
| Multi-layer structure (7 layers);  (This work) | | **High abs.** | | | **High abs.** | | **Low ε (0.270)** | **Low ε (0.042)** | **Low ε (0.218)** | | | **High ε (0.742)** | **High ε (0.473)** |
|  | Δ: for laser stealth | |  | *: for external lights at low temperatures | | | | | |  | /: not demonstrated | | |

**References**

1 Pan M, Huang Y, Li Q, Luo H, Zhu H, Kaur S *et al.* Multi-band middle-infrared-compatible camouflage with thermal management via simple photonic structures. *Nano Energy* 2020; **69**: 104449.

2 Zhu H, Li Q, Tao C, Hong Y, Xu Z, Shen W *et al.* Multispectral camouflage for infrared, visible, lasers and microwave with radiative cooling. *Nat Commun* 2021; **12**: 1805.

3 Lee N, Lim J-S, Chang I, Lee D, Cho HH. Transparent Metamaterials for Multispectral Camouflage with Thermal Management. *Int J Heat Mass Transf* 2021; **173**: 121173.

4 Park C, Kim J, Hahn JW. Integrated Infrared Signature Management with Multispectral Selective Absorber via Single‐Port Grating Resonance. *Adv Opt Mater* 2021; **9**: 2002225.

5 Ding D, He X, Liang S, Wei W, Ding S. Porous Nanostructured Composite Film for Visible-to-Infrared Camouflage with Thermal Management. *ACS Appl Mater Interfaces* 2022; **14**: 24690–24696.

6 Huang J, Wang Y, Yuan L, Huang C, Liao J, Ji C *et al.* Large‐Area and Flexible Plasmonic Metasurface for Laser–Infrared Compatible Camouflage. *Laser Photonics Rev* 2023; **17**: 2200616.

7 Kim J, Park C, Hahn JW. Metal–Semiconductor–Metal Metasurface for Multiband Infrared Stealth Technology Using Camouflage Color Pattern in Visible Range. *Adv Opt Mater* 2022; **10**: 2101930.

8 Wu Y, Luo J, Pu M, Liu B, Jin J, Li X *et al.* Optically transparent infrared selective emitter for visible-infrared compatible camouflage. *Opt Express* 2022; **30**: 17259.
